# Segmentation-based registration of ultrasound volumes for glioma resection in image-guided neurosurgery

**DOI:** 10.1007/s11548-019-02045-6

**Published:** 2019-08-07

**Authors:** Luca Canalini, Jan Klein, Dorothea Miller, Ron Kikinis

**Affiliations:** 1 Fraunhofer MEVIS, Institute for Digital Medicine, Bremen, Germany; 2 University of Bremen, Bremen, Germany; 3 Department of Neurosurgery, University Hospital Knappschaftskrankenhaus, Bochum, Germany; 4 Surgical Planning Laboratory, Brigham and Women’s Hospital, Harvard Medical School, Boston, USA

**Keywords:** Ultrasound, Image registration, Image segmentation, Convolutional neural network, Image-guided surgery

## Abstract

**Purpose:**

In image-guided surgery for glioma removal, neurosurgeons usually plan the resection on images acquired before surgery and use them for guidance during the subsequent intervention. However, after the surgical procedure has begun, the preplanning images become unreliable due to the brain shift phenomenon, caused by modifications of anatomical structures and imprecisions in the neuronavigation system. To obtain an updated view of the resection cavity, a solution is to collect intraoperative data, which can be additionally acquired at different stages of the procedure in order to provide a better understanding of the resection. A spatial mapping between structures identified in subsequent acquisitions would be beneficial. We propose here a fully automated segmentation-based registration method to register ultrasound (US) volumes acquired at multiple stages of neurosurgery.

**Methods:**

We chose to segment sulci and falx cerebri in US volumes, which remain visible during resection. To automatically segment these elements, first we trained a convolutional neural network on manually annotated structures in volumes acquired before the opening of the dura mater and then we applied it to segment corresponding structures in different surgical phases. Finally, the obtained masks are used to register US volumes acquired at multiple resection stages.

**Results:**

Our method reduces the mean target registration error (mTRE) between volumes acquired before the opening of the dura mater and during resection from 3.49 mm (± 1.55 mm) to 1.36 mm (± 0.61 mm). Moreover, the mTRE between volumes acquired before opening the dura mater and at the end of the resection is reduced from 3.54 mm (± 1.75 mm) to 2.05 mm (± 1.12 mm).

**Conclusion:**

The segmented structures demonstrated to be good candidates to register US volumes acquired at different neurosurgical phases. Therefore, our solution can compensate brain shift in neurosurgical procedures involving intraoperative US data.

## Introduction

In brain surgery for tumor removal, neurosurgeons usually plan the intervention on pre-surgical images. The most widely used modality for neurosurgery planning is magnetic resonance imaging [1, 2, 3]. To help physicians with the resection, neuronavigation systems can be used to link preplanning data positions to patient’s head locations. By tracking fiducial markers placed on the patient’s skull and surgical tools, an optical system computes an image-to-patient transformation. Consequently, by pin-pointing an intracranial location, neurosurgeons can obtain the same position in the preplanning images. However, initialization inaccuracies of the neuronavigation system may invalidate the image-to-patient transformation, affecting the quality of these images since the beginning of the resection [4]. Additionally, after resection starts, the preplanning data become even more unreliable due to the brain shift phenomenon: Structures observed in preplanning images don't remain in the same conformation and position during tumor removal [4]. As a consequence, the probability that pathological elements are missed increases, reducing the survival rates of the operated patients [5, 6]. To overcome this problem, intraoperative images can be acquired [7]: They provide an updated view of the ongoing procedure and hence compensate the brain shift effects. A solution is represented by intraoperative magnetic resonance imaging (iMRI) [8]. It is demonstrated to be a good option [9] since its high image quality provides good contrast in anatomical tissue even during the resection [10]. However, the high costs of iMRI and the architectural adaptations required in the operating room seem to prevent this modality from being deployed more widely. A valid alternative is given by intraoperative ultrasound (iUS) [11, 12, 13]. Some authors reported that for certain grades of glioma, iUS is equal or even superior to iMRI in providing good contrast between tumor and adjacent tissues [14, 15]. Moreover, US represents a lower-cost solution compared to MRI. In our work, we focus on intraoperative 3D ultrasound used in neurosurgical procedures.

The more the resection advances, the more the initial acquisition of iUS becomes unreliable due to increased brain shift effects. Therefore, an update of the intraoperative imaging may be required. In [16], the authors acquired US volumetric data in subsequent phases of glioblastoma resections in 19 patients and compared the ability to distinguish tumor from adjacent tissues at three different steps of the procedure. According to their observations, the 3D images acquired after opening the dura, immediately before starting the resection (we indicate this phase as *before resection*), are highly accurate for delineating tumor tissue. This ability reduces *during resection*, i.e., after that most of the resection has been performed but with residual tumor, and *after resection*, i.e., when all the detected residual tumor has been removed. In fact, the resection procedure itself is responsible for creating small air bubbles, debris and blood. Besides this, a blood clotting inducing material^1^ commonly used during neurosurgical procedures causes several image artefacts [14, 17]. Successive studies regarding other types of tumor resection confirmed the degradation of image quality in US during resection [18]. Therefore, it would be helpful to combine US images acquired during and after resection with higher-quality data obtained before resection. Such a solution may also be beneficial to improve the registration of intraoperative data with higher-quality preplanning MRI images. In fact, instead of combining directly degraded US data with preplanning imaging, it would be useful to register first the pre-surgical MRI data with US volumes acquired before resection, in which few anatomical modifications occurred. Afterward, intraoperative US data acquired at the first stage of the surgery (which therefore has a higher quality) may be registered to subsequent US acquisitions, and then the preplanning data could be registered to those by utilizing a two-step registration [19]. In this context, neuronavigation systems could be used to co-register intraoperative images acquired at different surgical phases. However, these devices are prone to technical inaccuracies, which affect the registration procedure from the beginning of the resection [4]. Moreover, the available neuronavigation systems usually offer only a rigid registration, which is not sufficient to address anatomical changes caused by brain shift. In our work, we propose a deformable method to improve the registration of US volumes acquired at different stages in brain surgery.

Few solutions have been proposed to improve the US–US registration during tumor resection in neurosurgery. In [20], the authors studied the performance of the entropy-based similarity measures joint entropy (JE), mutual information (MI) and normalized mutual information (NMI) to register ultrasound volumes. They conducted their experiments with two volumes of an US calibration phantom and two volumes of real patients, acquired before the opening the dura mater. Different rigid transformations were applied on each volume, and the target registration error (TRE) was used as evaluation metric. The accuracy of the registration was examined by comparing the induced transformation to move the original images to the deformed ones, with the transformation defined by the entropy-based registration method. In both of the datasets, NMI and MI outperformed JE. In another work [21], the same authors developed a non-rigid registration based on free-form deformations using B-splines and using normalized mutual information as a similarity measure. Two datasets of patients were used, where for each case a US volume was acquired before the opening of the dura, and one after (but prior to start of tumor resection). To assess the quality of the registration, the correlation coefficient was computed within the overlap of both volumes and before and after registration. Furthermore, these authors segmented the volumetric extension of the tumor with an interactive multiscale watershed method and measured the overlap before and after the registration. One limitation of the aforementioned two studies is that no experiment is conducted on volumes acquired at different stages of the surgical procedure, but only before the resection actually begins. In a real scenario, neurosurgeons use intraoperative data to find residual tumor after a first resection, which is conducted after the opening of the dura mater.

One of the first solutions to register US data obtained at subsequent surgical phases utilized an intensity-based registration method to improve the visualization of volumetric US images acquired before and after resection [22]. The results are computed for 16 patients with different grades of brain supratentorial tumor and located in various lobes. Half of the cases were first operations, and half were re-operations. Pre-resection volumes were acquired on the dura mater, or either directly on the cortex (or tumor) or on a dura repair patch. The post-resection ultrasound was used to find any residual tumor. The authors used mutual information as similarity measure for a rigid registration. In the further non-rigid transformation, the correlation coefficient objective function was used. To correctly evaluate their findings, for each of the 16 cases, a neuroradiologist chose 10 corresponding anatomic features across US volumes. The initial mean Euclidean distance of 3.3 mm was reduced to 2.7 mm with a rigid registration, and to 1.7 mm with the non-rigid registration. The quality of the alignment of the pre- and post-resection ultrasound image data was also visually assessed by a neurosurgeon. Afterward, an important contribution to neurosurgical US–US registration came by the release of the *BITE* dataset [23], in which pre- and post-resection US data are publicly available with relative landmarks to test registration methods. One of the first studies involving BITE dataset came from [17]. The authors proposed an algorithm for non-rigid *REgistration of ultraSOUND* images (*RESOUND*) that models the deformation with free-form cubic B-splines. Normalized cross-correlation was chosen as similarity metric, and for optimization, a stochastic descendent method was applied on its derivative. Furthermore, they proposed a method to discard non-corresponding regions between the pre- and post-resection ultrasound volumes. They were able to reduce the initial mTRE from 3.7 to 1.5 mm with a registration average time of 5 s. The same method has been then used in [19]. In a compositional method to register preoperative MRI to post-resection US data, they applied the RESOUND method to register first pre- and post-resection US images. In another solution [24], the authors aimed to improve the RESOUND algorithm. They proposed a symmetric deformation field and an efficient second-order minimization for a better convergence of the method. Moreover, outlier detection to discard non-corresponding regions between volumes is proposed. The BITE mean distance is reduced to 1.5 mm by this method. Recently, another method to register pre- and post-resection US volumes was proposed by [25]. The authors presented a landmark-based registration method for US–US registration in neurosurgery. Based on the results of 3D SIFT algorithm [26], images features were found in image pairs and then used to estimate dense mapping through the images. The authors utilized several datasets to test the validity of this method. A private dataset of nine patients with different types of tumor was acquired, in which 10 anatomical landmarks were selected per case, in both pre- and post-resection volumes: For this set, they were able to reduce the mTRE from 3.25 mm to 1.54 mm. Then, they applied the same method on the BITE dataset and reduced the initial mean error to 1.52 mm. Moreover, they tested their approach on the more recent RESECT dataset [14]. By using the same method on the pre- and post-resection volumes, the mTRE was reduced from 3.55 to 1.49 mm.

Our solution proposes a segmentation-based registration approach to register US volumes acquired at different stages of neurosurgical procedures and compensate brain shift. A few approaches already applied segmentation methods on US data to then register MRI and iUS [27, 28]. Our solution represents the first segmentation-based method aimed at US–US volumes registration. Our approach includes a deep-learning-based method, which automatically segments anatomical structures in subsequent US acquisitions. We chose to segment the hyperechogenic structures of the sulci and falx cerebri, which remain visible during the resection and thus represent good corresponding elements for further registration. In the following step, parametric and nonparametric methods use the generated masks to register US volumes acquired at different surgical stages. Our solution reduces the initial mTRE for US volumes acquired at subsequent acquisitions in both RESECT and BITE datasets.

## Materials and methods

### Datasets

We used two different public datasets to validate our segmentation-based registration method. Most of our experiments are conducted on the RESECT dataset [14], including clinical cases of low-grade gliomas (Grade II) acquired on adult patients between 2011 and 2016 at St. Olavs University Hospital, Norway. There is no selection bias, and the dataset includes tumors at various locations within the brain. For 17 patients, B-mode US-reconstructed volumes with good coverage of the resection site have been acquired. No blood clotting agent, which causes well-known artefacts, is used. US acquisitions are performed at three different phases of the procedure (before resection, during and after resection), and different US probes have been utilized. This dataset is designed to test intra-modality registration of US volumes and two sets of landmarks are provided: one to validate the registration of volumes acquired before, during and after resection, and another set that increases the number of landmarks between volumes obtained before and during resection. Regarding both sets, the reference landmarks are taken in the volumes acquired before resection and then are utilized as references to select the corresponding landmarks in US volumes acquired during and after tumor removal. In the RESECT dataset, landmarks have been taken in the proximity of deep grooves and corners of sulci, convex points of gyri and vanishing points of sulci. The number of landmarks of the first and second sets can be, respectively, found in the second column of Tables 4 and 5.

In addition to RESECT volumes, BITE dataset is also utilized to test our registration framework [23]. It contains 14 US-reconstructed volumes of 14 different patients with an average age of 52 years old. The study includes four low-grade and ten high-grade gliomas, all supratentorial, with the majority in the frontal lobe (9/14). For 13 cases, acquisitions are obtained before and after tumor resection. Ten homologous landmarks are obtained per volume, and initial mTRE are provided. The quality of BITE acquisitions is lower with respect to RESECT dataset, mainly because blood clotting agent is used, creating large artefacts [14].

### Methods

We used MeVisLab^2^ for implementing (a) an annotation tool for medical images, (b) a 3D segmentation method based on a CNN and (c) registration framework for three-dimensional data.

#### Manual segmentation of anatomical structures

The first step of our method consists of the 3D segmentation of anatomical structures in different stages of US acquisitions. Both RESECT and BITE datasets are used to test registration algorithms and no ground truth is provided for validating segmentation methods. Therefore, we decided to conduct a manual annotation of the structures of interest in the US volumes acquired before resection of RESECT dataset. Pathological tissue was excluded from the manual annotation since it is progressively removed during resection and correspondences could not be found in volumes acquired at subsequent stages. On the contrary, we focused on other *hyperechogenic* (with an increased response—echo—during ultrasound examination) elements such as the sulci and falx cerebri. We consider these elements valid correspondences because the majority of them has a high chance to remain visible in different stages of the procedure.

**Fig. 1:**
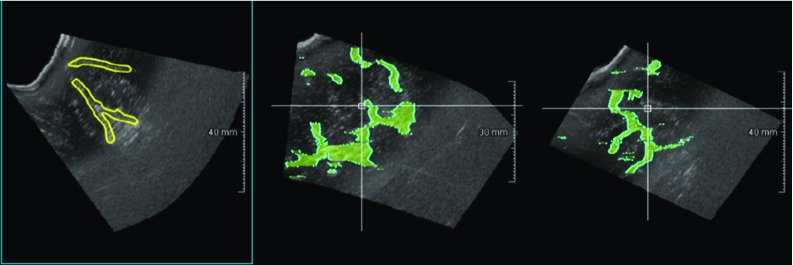
Web-based annotation tool. While contouring the structures of interest on the axial view (yellow line in the left frame), the segmentation process can be followed in real time on the other two views of US volumes. The annotation tool is accessible by common web browsers, and it has been used to obtain and then review the manual annotation

The manual segmentations were performed on a web-based annotation tool. As shown in Fig. 1, each RESECT volume can be simultaneously visualized on three different projections planes (axial, sagittal and coronal). The segmentation task is accomplished by contouring each structure (yellow contour in the first frame of Fig. 1) of interest on the axial view. The drawn contours are then projected onto the other two views (blue overlay in the second frames of Fig. 1) so that a better understanding of the segmentation process is possible by observing the structures in different projections. The annotation process can be accomplished very easily and smoothly, and 3D interpolated volumes can be then obtained by rasterizing the drawn contours. As shown in Fig. 1, the contours are well defined in the axial view but several elements are not correctly included if considering the other two views. This is a common issue that we found in our annotation, which would require much time and effort to be corrected. However, we decided to have a maximum annotation time of 2 h per volume. The obtained masks correctly include the major structures of interest, but some elements such as minor sulci are missing. Despite the sparseness of our dataset, we expect our training set to be good enough to train our model to segment more refined structures of interest [29, 30].

The manual annotation was performed by the main author of this work (L.C.), who has two years of experience in medical imaging and almost one year in US imaging for neurosurgery. Then, a neurosurgeon with many years of experience in the use of US modality for tumor resection reviewed and rated the manual annotations, by taking into account the sparseness of the dataset. According to the defined criteria, each volume could be rated with a point between 4 and 1. More precisely, a point equal to 1 means that the main structures (falx cerebri and major sulci) are correctly segmented, and only minor changes should be done to exclude parts of no interest (i.e., slightly over-segmented elements). A point equal to 2 indicates that the main structures are correctly segmented, but major corrections should be done to exclude structures of no interest. A point equal to 3 indicates that main structures were missed in the manual annotations, which, however, are still acceptable. A score of 4 means that a lot of major structures are missing; therefore, that annotation for the volume of interest cannot be accepted. The neurosurgeon evaluated the annotations by looking at the projected structures on the sagittal and coronal views of the drawn contours. Table 1 shows the results of the rating process for the volumes of interest.

**Table 1:** Rating of the manual annotations

Volumes	1	2	3	4	6	7	12	14	15	16	17	18	19	21	24	25	27
Ranking	2	2	3	2	2	3	2	3	2	3	2	2	2	2	2	2	2

After the contours of the main structures of interest were manually drawn, the neurosurgeons rated them according to criterion defined in the session “Manual segmentation of anatomical structures”. The criterion is defined taking into account the sparseness of the manual annotations. A point equal to 4 is given to the annotations where many of the main structures of interest are missing. On the contrary, if minor structures of interest (i.e., minor sulci) are missing but the major ones are correctly included, the best point of 1 is given

#### Segmentation

A convolutional neural network aimed for a volumetric segmentation is trained on the manual annotations. We utilized the original 3D U-net [29] architecture, in which few modifications were made with respect to the original implementation: (a) The analysis and synthesis paths have two resolution steps and (b) before each convolution layer of the upscaling path a dropout with a value of 0.4 is used in order to prevent the network from overfitting. The training is conducted with a patch size of (30,30,30), padding of (8,8,8) and a batch size of 15 samples. The learning rate was set to 0.001, and the best model saved according to best *Jaccard* index computed on 75 samples every 100 iterations. The architecture modifications, as well as the training parameters, were chosen by conducting several experiments and selecting those providing the best results. As training, validation and test sets, we split the seventeen volumes acquired before resection, which we annotated in the manual annotation. The split has been done as follows: The training set includes the volumes from 1 to 15, the validation one the volumes from 16 to 21 and the test one the volumes 24, 25, 27.

After having found the best model to segment anatomical structures in pre-resection US volumes, we applied it to segment ultrasound volumes acquired at different surgical phases.

#### Registration

The masks automatically segmented by our trained model are used to register US volumes. The proposed method is a *variational* image registration approach based on [31]: The registration process can be seen as an iterative optimization algorithm where the search of the correct registration between two images corresponds to an optimization process aimed at finding a global minimum of an objective function. The minimization of the objective function is performed according to the discretize-then-optimize paradigm [31]: The discretization of the various parameters is followed by their optimization. The objective function to be minimized is composed of a *distance measure*, which quantifies the similarity between the deformed template image and the reference one, and a *regularizer*, which penalizes undesired transformations. In our approach, the binary 3D masks generated by the previous step are used as input for the registration task, which can be seen as mono-modality intensity-based problem. Therefore, we chose the sum of squared differences (SSD) as a similarity measure, which is usually suggested to register images with similar intensity values. Moreover, to limit the possible transformations in the deformable step, we utilized the elastic regularizer, which is one of the most commonly used [31]. In our method, the choice of the optimal transformation parameters has been conducted by using the quasi-Newton l-BGFS [32], due to its speed and memory efficiency. The stopping criteria for the optimization process were empirically defined: the minimal progress, the minimal gradient and the relative one, the minimum step length were set equal to 0.001, and the maximum number of iterations equal to 100.

Our registration method aims to provide a deformable solution to compensate for anatomical changes happening during tumor resection. As commonly suggested for methods involving non-rigid registration tasks [31], the proposed solution includes an initial parametric registration used then to initialize the nonparametric one. First of all, the parametric approach utilizes the information provided by the optical tracking systems as an initial guess. Based on this pre-registration, a two-step approach is conducted, including a translation followed then by a rigid transformation. In this stage, to speed the optimization process, the images are registered at a resolution one-level coarser compared to the original one. Then, the information computed during the parametric registration is utilized as the initial condition for the nonparametric step. In this stage, to reduce the chance to reach a local minimum, a multilevel technique is introduced: the images are registered at three different scales, from a third-level to one-level coarser. As output of the registration step, the deformed template image is provided.

## Evaluation

### Segmentation

In Table 1, we can see that no annotation received the best score of 1, but all of them have some imperfections. However, none of the manually annotated masks was scored with 4. Consequently, we can consider our annotations as a sparse ground truth in which only the main hyperechogenic structures of interest are included. Regarding this, CNNs trained on a sparse dataset already proved to be able to segment more refined and numerous structures respect to the sparse training set [29, 30]. Therefore, we expect our annotations to be good enough to train the CNN model in order to generate meaningful structures for guiding the further registration step. In fact, the registration step will give an important feedback about the quality of the generated masks: For our purposes, the segmented structures are meaningful if they correctly guide the registration method. In addition to this, an analysis of the segmentation results will be provided, as described in the following section.

Regarding US volumes acquired before resection, no ground truth is available for the structures not contained in the manual annotations. Consequently, the DICE coefficients are computed by including only the automatically segmented elements with correspondences to manual annotations and by discarding elements having no counterpart in manually annotated data. This measure is useful to verify whether the main structures of interest are correctly segmented by the trained model. As further information, we also provide the DICE coefficients computed without excluding any structure. These values would be useful for a deeper analysis of our algorithm but, as aforementioned, they may not be so indicative for our purposes due to the sparseness of our dataset. Furthermore, the automatically generated masks should also include more refined elements than the original ground truth. To verify this, a first visual assessment of the generated masks is performed. Moreover, the over-segmented elements are expected to have a mean intensity value as close as possible to the one of the manually annotated structures. To verify this, we compared the mean intensity values of the manual annotations and the automatically generated masks.

Regarding US volumes acquired during and after resection, no manual annotation was obtained, so no DICE index could be computed. Therefore, to be sure that structures of interest are correctly segmented, we show that the masks of the three stages of US data segmented by our trained model (a) are strongly correlated in terms of volume extension by computing the Pearson correlation coefficient and (b) include structures with a mean intensity value similar to the manual annotations. Secondly, we conduct a visual inspection of the results, which is helpful to verify whether or not corresponding anatomical structures are segmented in these stages.

Given the fact that our annotations are not publicly available, only a qualitative comparison is made with respect to other methods which also proposed a US segmentation solution in the context of neurosurgery [27, 28, 30, 33].

### Registration

The transformations and deformation fields computed in the parametric and nonparametric step are then applied to the landmarks contained in the datasets. The TRE values before and after registration are provided per each patient, with the measure of the closest and farthest couple of points, and mean and standard deviation values are also given per each set of landmarks. A visual inspection of the registration results is also shown, in which the initial registration based on the information of the optical tracker can be compared with the results obtained by our method. Moreover, a comparison with previous solutions is provided. Regarding this, some methods have been proposed to register BITE volumes [17, 19, 24, 25], but none of them except one [25] provided a generalized solution able to register volumes of both datasets (BITE and RESECT). On the contrary, our method provides an approach valid for both two datasets. For the RESECT dataset, the authors of [25] proposed a solution only for volumes acquired before and after resection. Our approach is the first one to be applied to the volumes acquired before and during resection of RESECT dataset; therefore, no comparison is available for this specific set.

The *capture range* of our method is also computed. We define the capture range as the largest initial misalignment within which our algorithm still converges to a solution for 80% of the cases. To evaluate it, we started the registration from multiple starting misalignments and we checked whether or not the method converged to a solution. Then, we computed the value of the capture range by using as distance measure the mTRE computed on the available landmarks.

## Results

### Segmentation

**Fig. 2:**
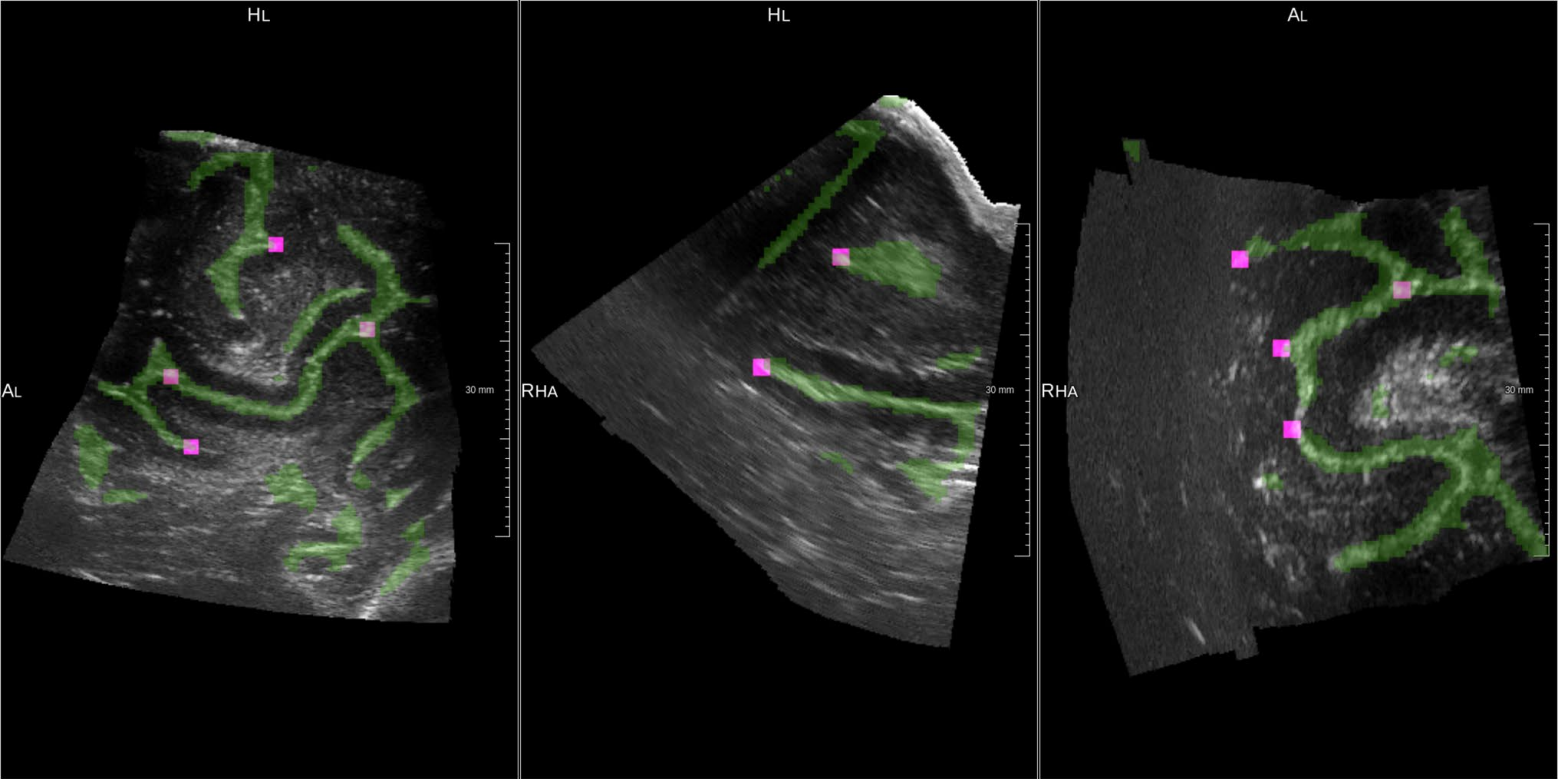
Segmentation and landmarks. Original intensity volumes where the generated masks (in green) and RESECT landmarks (purple squares) are overlaid. In RESECT dataset, landmarks have been taken in proximity of deep grooves and corners of sulci, convex points of gyri, and vanishing points of sulci. We chose to segment sulci falx cerebri, and therefore, we can see how the landmarks are closely located to the segmented structures

Figure 2 shows an example of a segmented structure in a volume acquired before resection. It can be seen that the generated masks cover the locations where landmarks were acquired. In fact, we decided to segment sulci and falx cerebri, which are the anatomical elements taken into account to acquire the majority of the landmarks in the RESECT dataset.

**Fig. 3:**
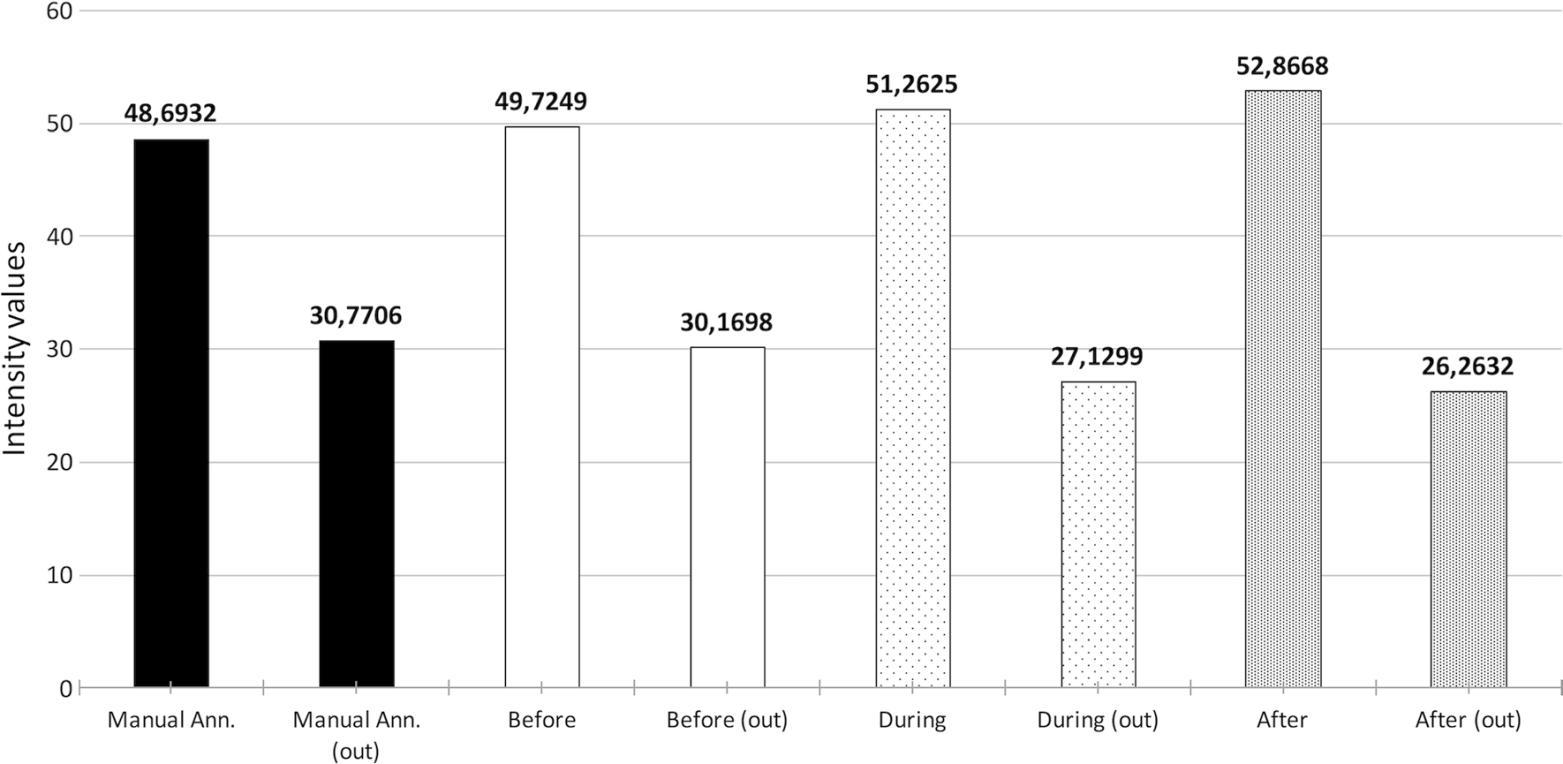
Intensity values of the masked ultrasound volumes. This graph presents the mean intensity values of the masked ultrasound volumes (first, third, fifth, seventh bars) acquired at the three stages, and the mean intensity values of the area excluded by the segmentation (second, fourth, sixth, eighth bars). For the volumes acquired before resection, volumes masked with manual annotation and elements segmented by the neural network are compared (first four bars). The masked volumes have in all the cases a similar mean value, higher than the excluded areas. This is meaningful since our elements of interest are the bright (hyperechogenic) structures in the US. On the contrary, the even numbered bars have a similar mean intensity value, lower than the chosen structures. We are not interested in hypoechogenic structures, with look darker in the US acquisitions

**Fig. 4:**
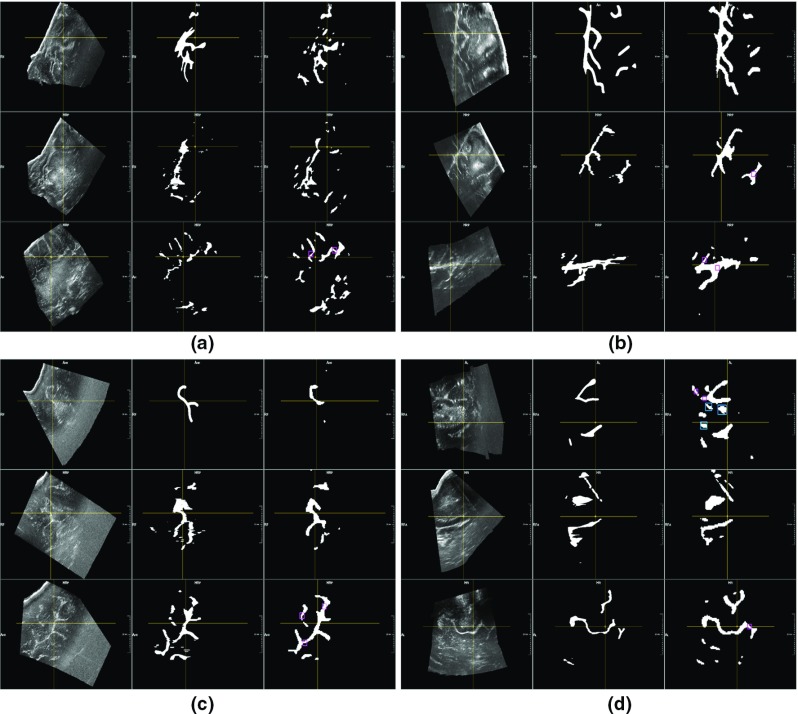
Segmentation of ultrasound volumes acquired before resection. In each example, the axial, sagittal and coronal views are shown in the first, second and third row, respectively. In the first column, the original ultrasound volume is exhibited, in the second column, the manual annotation performed on the axial view and projected in the other two views is shown, in the third column, the segmentation result obtained by the 3D U-net for the same volume of interest is displayed. In each example, a pointer (intersection of yellow crossing lines) highlights the same volume position in the three views. Our method correctly segments the main structures. Moreover, structures wrongly not included in the manual annotations are correctly detected by the trained neural network (purple squares). However, in image d, pathological tissue correctly excluded in the original masks is wrongly segmented by our method (blue squares in axial view)

Regarding US data acquired before resection, Table 2a provides the DICE coefficients computed between the manually segmented structures and the corresponding masks generated by our trained model. In Table 2b, the DICE coefficients for the whole set of generated masks (without excluding the elements not included in the manual annotation) are given. Furthermore, the first and third bars in Fig. 3 show that the structures automatically segmented in pre-resection volumes have a mean intensity value very similar to those chosen in the manual annotations. A similar consideration can be made for the elements considered as background (second and fourth bars in Fig. 3). Qualitative results also confirm this evidence. Figure 4 shows four examples of automatically generated masks in comparison with the corresponding manual annotations. In most of the cases, our method correctly segments refined elements which were not included in the manual annotation due to timing restriction see “Manual segmentation of anatomical structures”. Violet squares highlight some examples of these structures. Though, in several cases, the neural network wrongly segments pathological tissue which we excluded from the manual annotations (see blue squares in Fig. 4d).

**Table 2:** DICE coefficients for volumes acquired before surgery

Volumes	1	2	3	4	6	7	12	14	15	16	17	18	19	21	24	25	27
(a)																	
Dice %	68	62	57	76	71	56	78	76	78	61	62	70	70	63	74	68	69
(b)																	
Dice %	62	46	28	59	50	46	67	67	63	53	45	35	61	42	58	44	51

(a) Refers to the DICE coefficient computed by considering only the structures with a counterpart in the manual annotations. The method shows evidence of being able to properly segment the anatomical structures considered in the manual annotations. (b) Refers to the DICE values for the whole set of the automatically segmented structures

**Fig. 5:**
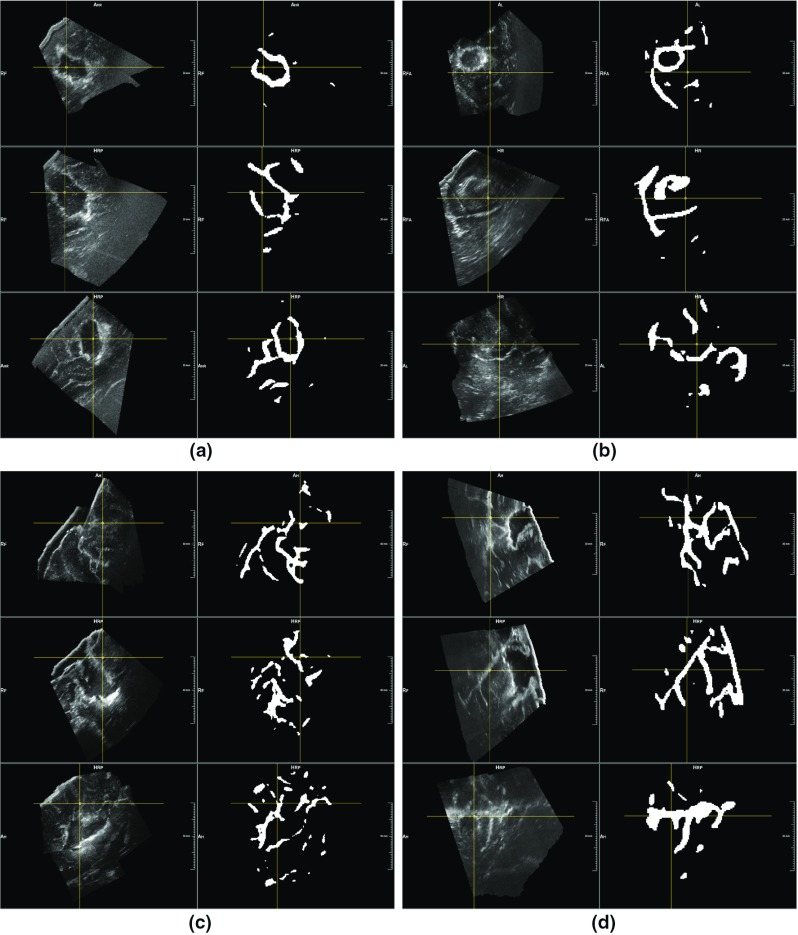
Segmentation of ultrasound volumes acquired during resection. After having trained the neural network on the stage before resection, we applied it to ultrasound volumes acquired during resection. This figure shows four examples of segmentation results, each containing one intensity volume together with the generated mask. It appears clear how the main hyperechogenic structures are correctly included in the segmentation

**Fig. 6:**
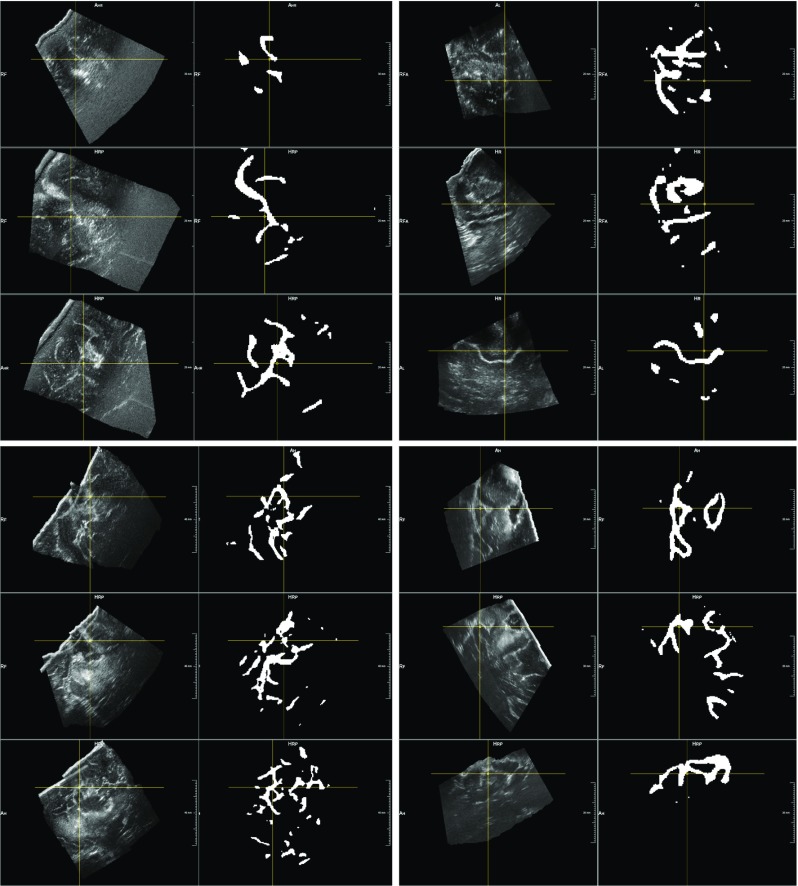
Segmentation of ultrasound volumes acquired after resection. After having trained the neural network on the stage before resection, we applied it to ultrasound volumes acquired after resection. This figure shows four examples of segmentation results, each containing one intensity volume together with the generated mask. It appears clear how the main hyperechogenic structures are correctly included in the segmentation. In the last two examples (second row), we see how resection cavities (appearing hyperechogenic on US) are segmented by the 3D U-net, even they have no counterparts in the pre-resection stage

For the volumes acquired during and after resection, a strong correlation exists between the extension of their masks segmented by the neural network and of the volumes before resection. In fact, the Pearson coefficient between the masks of US data acquired before and during resection has a value of 0.90, and a value of 0.91 for those of pre- and post-removal. As for the US data acquired before resection, Fig. 3 shows that the anatomical structures segmented at the different stages have a mean intensity similar to the manual annotation (last four bars). Therefore, we can state that our segmentation method, applied to volumes acquired at different stages, segment structures related to each other in terms of volumes extension and mean intensity values. Then, visual results in Figs. 5 and 6 confirm the evidence of the quantitative results, showing that our model trained on a stage of US correctly segments analogous elements in volumes acquired at different stages. However, qualitative results in Fig. 6 also show that our method often detects resection cavities, which have no corresponding structures in the pre-resection volumes.

### Registration

The mean time required by the registration tasks is given in Table 3, together with the mean time required by each volume to be segmented by the trained model. All experiments are made on a computer equipped with an Intel Core i7 and a GeForce GTX 1080 (8 GB).

**Table 3:** Mean time in seconds per task

Mean time (in s) per each task		
Segmentation (inference)	Total registration time (US before–during)	Total registration time (US during–after)
1.28	28.55	29.40

With *segmentation*, we indicate the inference process in which the trained model generates the mask of a volume given in input. The other two values are related to the registration tasks, including the time of both the parametric and nonparametric approaches

**Fig. 7:**
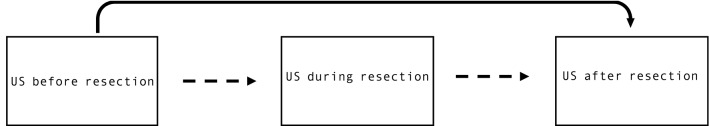
Registration of different US volume pairs. Instead of registering directly pre-resection US data with those after resection (continuous line), a two-step method (dotted arrows) is proposed by including the US volumes acquired during resection. The final transformation from before to after resection volumes is obtained by concatenating two different registrations results (US before resection to US during resection + US during resection to US after resection)

By relying on the automatically generated masks in the segmentation step, we registered the US volumes acquired at different surgical stages. First, the volumes acquired before and during resection are registered. Then, our algorithm is applied to volumes acquired during and after resection. The computed deformation fields are applied to the landmarks provided in the RESECT dataset, and the results after registration are shown in Table 4 (for volumes acquired before and during resection) and in Table 5 (volumes acquired before and after resection). Regarding the results in Table 5, the registration of the landmarks is performed by concatenating two different transformations: the one computed before–during US volumes together with the one for volumes acquired during and after resection (see Fig. 7 for a more detailed description).

**Table 4:** Registration errors on RESECT dataset

		Mean distance (range) in mm before versus during (RESECT dataset)		
Patient	Number of landmarks	Mean initial distance	Mean distance after parametric registration	Mean distance after nonparametric registration
1	34	2.32 (1.49–3.29)	0.93 (0.31–1.73)	0.89 (0.22–1.57)
2	16	3.10 (1.79–5.19)	1.54 (0.37–3.58)	1.69 (0.71–4.19)
3	17	1.93 (0.67–3.02)	1.20 (0.36–2.47)	1.14 (0.24–2.45)
4	19	4.00 (3.03–5.22)	0.89 (0.31–1.86)	0.83 (0.24–1.65)
6	21	5.19 (2.60–7.18)	1.95 (0.63–3.71)	1.80 (0.58–3.61)
7	22	4.69 (0.94–8.16)	2.50 (1.24–5.78)	2.39 (1.15–5.86)
12	24	3.39 (1.74–4.81)	1.57 (0.43–3.20)	1.58 (0.44–3.36)
14	22	0.71 (0.42–1.59)	0.52 (0.09–1.15)	0.52 (0.12–0.93)
15	21	2.04 (0.85–2.84)	0.80 (0.28–1.44)	0.73 (0.18–1.31)
16	19	3.19 (1.22–4.53)	1.52 (0.95–2.21)	1.40 (0.75–2.43)
17	17	6.32 (4.65–8.07)	2.93 (1.67–4.46)	2.51 (1.14–4.03)
18	23	5.06 (1.55–7.44)	1.75 (0.70–3.04)	1.29 (0.46–2.81)
19	21	2.06 (0.42–3.40)	1.93 (0.20–3.19)	1.33 (0.48–2.67)
21	18	5.10 (3.37–5.94)	1.27 (0.19–3.53)	1.22 (0.19–3.46)
24	21	1.76 (1.16–2.65)	0.89 (0.18–2.17)	0.81 (0.08–2.07)
25	20	3.60 (2.19–5.02)	3.56 (2.09–5.14)	2.27 (1.04–3.92)
27	16	4.93 (3.61–7.01)	0.77 (0.24–1.35)	0.71 (0.19–1.26)
		Mean value	Mean value	Mean value
		3.49 ± 1.55	1.56 ± 0.82	1.36 ± 0.61

Mean registration errors between ultrasound volumes acquired before and during resection. Original distances are compared to the results obtained with our segmentation-based registration

**Table 5:** Registration errors on RESECT dataset

		Mean distance (range) in mm before vs. after (RESECT Dataset)		
Patient	Number of landmarks	Mean initial distance	Mean distance after parametric registration	Mean distance after nonparametric registration
1	13	5.80 (3.62–7.22)	2.69 (0.93–4.08)	2.67 (0.75–4.18)
2	10	3.65 (1.71–6.72)	2.32 (0.90–4.25)	2.18 (0.55–3.93)
3	11	2.91 (1.53–4.30)	1.63 (0.82–2.48)	1.53 (0.82–2.25)
4	12	2.22 (1.25–2.94)	1.05 (0.46–1.95)	1.06 (0.30–2.05)
6	11	2.12 (0.75–3.82)	1.91 (0.47–3.06)	1.88 (0.24–2.93)
7	18	3.62 (1.19–5.93)	2.29 (0.92–4.13)	2.08 (0.70–3.93)
12	11	3.97 (2.58–6.35)	1.60 (0.54–4.73)	1.44 (0.61–4.51)
14	17	0.63 (0.17–1.76)	0.63 (0.11–1.84)	0.57 (0.09–1.52)
15	15	1.63 (0.62–2.69)	0.85 (0.12–2.13)	0.88 (0.23–2.38)
16	17	3.13 (0.82–5.41)	2.40 (0.61–4.70)	2.14 (0.79–4.35)
17	11	5.71 (4.25–8.03)	3.82 (2.36–6.68)	3.40 (1.91–6.28)
18	13	5.29 (2.94–9.26)	2.19 (1.14–4.32)	1.51 (0.65–2.92)
19	13	2.05 (0.43–3.24)	4.00 (1.42–14.27)	3.97 (0.91–15.29)
21	9	3.35 (2.34–5.64)	1.23 (0.29–3.20)	1.18 (0.28–3.16)
24	14	2.61 (1.96–3.41)	0.86 (0.18–2.26)	0.79 (0.13–2.02)
25	12	7.61 (6.40–10.25)	5.75 (4.39–8.34)	3.88 (2.74–6.07)
27	12	3.98 (3.09–4.82)	3.77 (2.22–5.10)	3.76 (2.24–5.30)
		Mean value	Mean value	Mean value
		3.54 ± 1.75	2.29 ± 1.37	2.05 ± 1.12

Other methods	mTRE after registration
[25]	1.49 mm

Mean registration errors between ultrasound volumes acquired before and after resection. Original distances are compared to the results obtained with our segmentation-based registration. Moreover, a comparison is made with a previous method proposed to solve this task

**Fig. 8:**
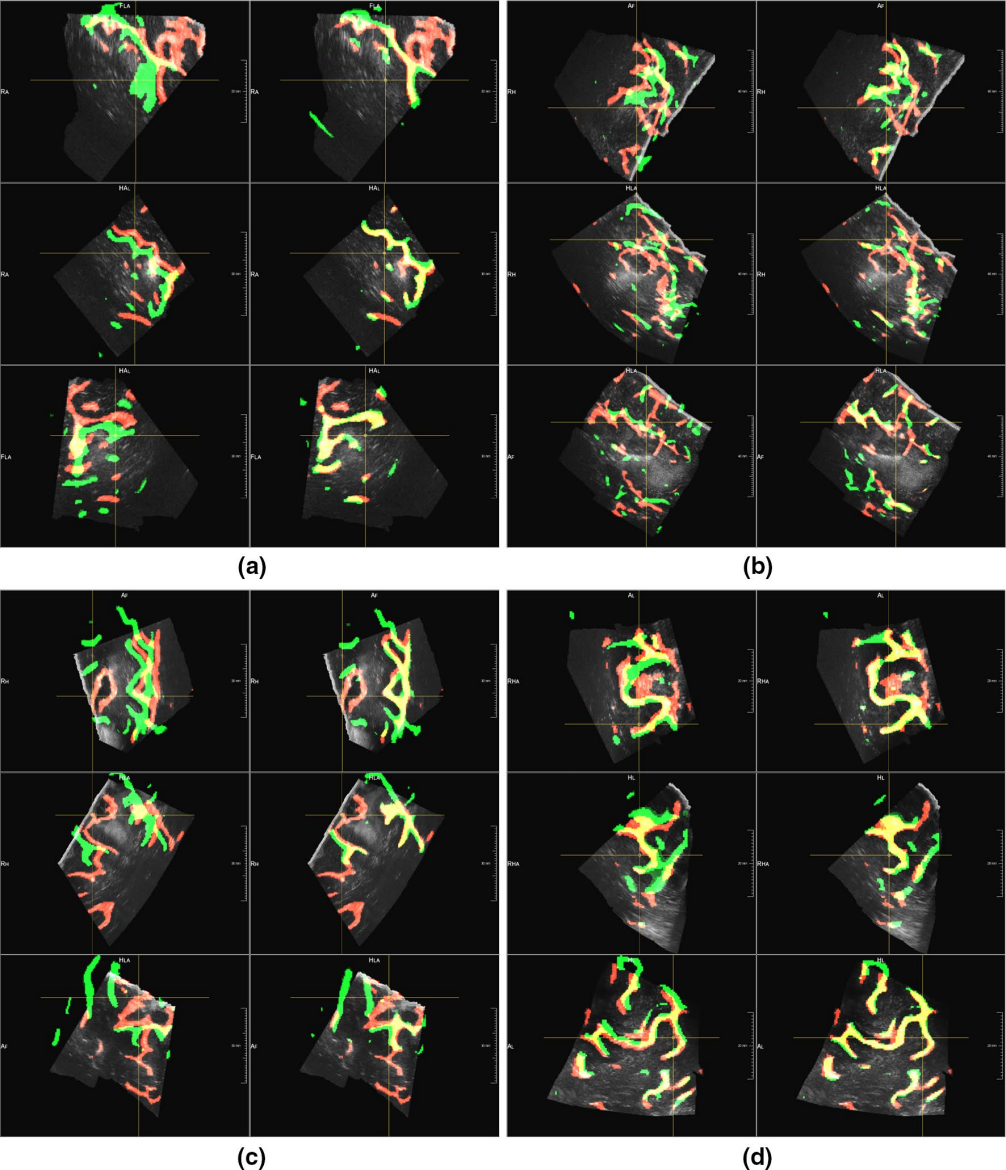
Registration results for before and during resection volumes. The images show four examples of registration by combining fixed volumes (during resection) with its segmented structures (in red) and the segmented elements of moving volumes acquired before resection (in green). In the first column of each example, we show the segmentation overlay according to the original information. The second column displays the overlay of the segmented structures after registration. By highlighting in yellow the correct overlap of segmented structures, we can see how the structures are more aligned after the performed registration

**Fig. 9:**
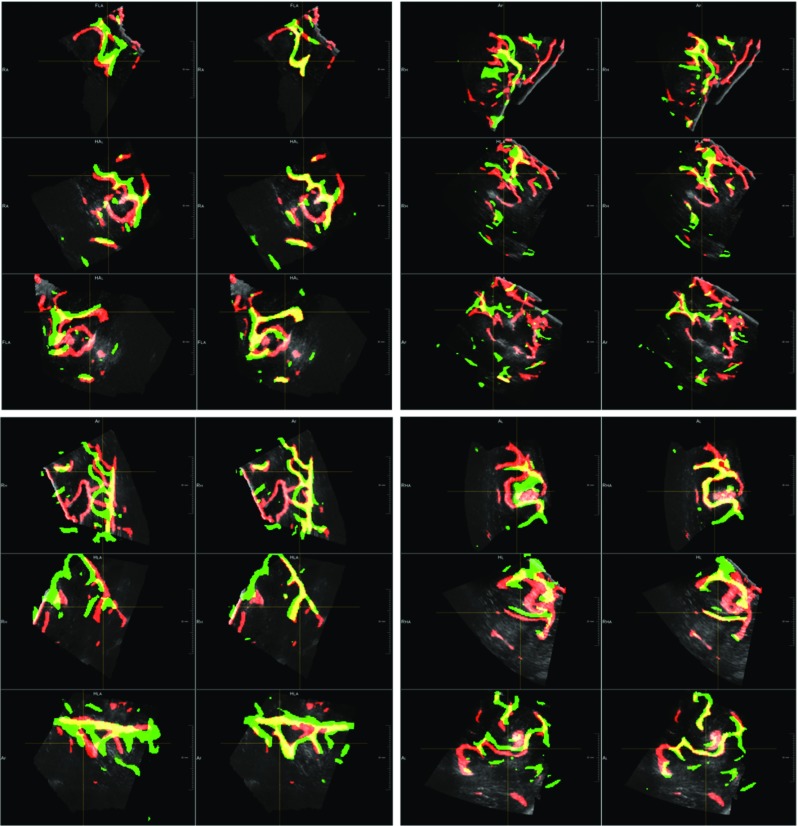
Registration results for before and after resection volumes. These images show four examples of registration by combining fixed volumes (after resection) with its segmented structures (in red) and the segmented elements of moving volumes acquired before resection (in green). In the first columns of each example, we show the segmentation overlay according to the original information. The second column displays the overlay of the segmented structures after registration. By highlighting in yellow the correct overlap of segmented structures, we can see how the structures are more aligned after the performed registration

As it can be seen in both tables, both parametric and nonparametric methods reduce the initial mean registration errors provided in the RESECT dataset. In Table 4, it can be noticed that the proposed methodology improves the initial mTRE more than 2 mm, by decreasing the mean errors for each patient. For the second registration tasks, our method reduces the mean registration error by nearly 1.5 mm (Table 5). Visual examples provided in Figs. 8 and 9 also confirm the numerical results. The images show the fixed volumes with the related segmentation (in red), together with the mask of the moving volumes (in green). By comparing the overlay before and after registration, we highlight the registration improvements by coloring in yellow the correct overlay of the two masks. Regarding the results on the RESECT dataset, only those obtained for volumes acquired before and after resection can be compared with another solution [25] (see Table 5).

Our segmentation-based registration method is then applied on BITE dataset, directly registering volumes acquired before and after resection. The results are available in Table 6, with a comparison to previously proposed solutions (last section of Table 6). As it can be seen, also for this dataset the initial mTRE is reduced by both parametric and nonparametric registration approaches.

**Table 6:** Registration errors on BITE dataset

Mean distance (range) in mm before vs. after (BITE dataset)			
Patient	Mean initial distance	Mean distance after parametric registration	Mean distance after nonparametric registration
2	2.30 (0.57–5.42)	1.97 (0.69–4.84)	1.70 (0.51–4.70)
3	3.40 (0.0–5.09)	3.12 (0.43–4.73)	1.45 (0.25–3.48)
4	4.60 (2.96–5.88)	3.62 (2.43–4.81)	2.20 (1.02–3.62)
5	4.11 (2.58–5.52)	3.68 (2.39–5.04)	1.32 (0.46–2.96)
6	2.26 (1.36–3.10)	1.33 (0.56–2.00)	1.17 (0.30–1.64)
7	3.87 (2.60–5.07)	1.27 (0.83–2.36)	1.17 (0.75–1.75)
8	2.51 (0.67–3.93)	2.11 (0.54–3.35)	2.18 (1.05–3.43)
9	2.21 (1.00–4.59)	1.99 (0.64–4.52)	1.95 (0.58–4.54)
10	3.86 (0.98–6.68)	3.81 (1.91–6.22)	3.43 (1.53–5.69)
11	2.74 (0.44–8.22)	2.74 (0.51–7.55)	2.39 (0.35–7.42)
12	10.54 (7.85–13.04)	10.88 (8.28–13.34)	11.08 (8.36–13.72)
13	1.62 (1.33–2.21)	0.73 (0.39–1.43)	0.75 (0.30–1.67)
14	2.19 (0.59–3.99)	1.60 (0.60–2.81)	1.43 (0.41–2.24)
	Mean value	Mean value	Mean value
	3.55 ± 2.28	2.98 ± 1.80	2.48 ± 2,67

Other methods	mTRE after registration
[17]	1.50 mm
[19]	1.50 mm
[24]	1.50 mm
[25]	1.54 mm

Mean registration errors between ultrasound volumes acquired before and after resection. In this dataset, a fixed number of markers (10 per case) is provided. Original distances are compared to the results obtained with our segmentation-based registration. Moreover, a comparison with previously proposed solutions (with corresponding mTREs) is provided

The value of the capture range of our method is equal to 6.25 mm.

## Discussion

The manual annotations, even if sparse, are good enough to train the CNN model to segment the anatomical structures of interest, as shown by the DICE coefficients in Table 2. Moreover, Fig. 4 shows that automatically generated segmentations are more precise than the manual annotations, with a better contours refinement and larger number of identified structures. However, some pathological tissues are wrongly segmented by our method (see Fig. 4d). This may be due to the fact that in US data the glioma of grade II appears as hyperechogenic structures, with an intensity similar to the elements of interest. In future work, we could consider to separately segment pathological tissue and then exclude it during registration. A similar consideration can be made for the resection cavities in volumes acquired during and after resection, which appear as bright as sulci and are wrongly segmented by the proposed method (Fig. 6). Furthermore, from a qualitative comparison with other segmentation methods involving US data, we can highlight some advances of our approach. First of all, with respect to [27, 33], a higher number of anatomical structures are included in our manual annotations. Therefore, the potential range of clinical scenarios in which our method could be applied might be wider. Secondly, a trained neurosurgeon has clinically validated the manual annotations (Table 1). This is not the case for other segmentation-based methods [30, 28], in which no precise rating of the manual masks is provided.

The second important contribution of this work is the registration of US volumes acquired at different surgical stages. First of all, the segmentation method gives evidence of being able to generate meaningful masks to guide the registration task. In fact, the proposed registration method is able to reduce the mTREs of three sets of volumes from two different datasets (Table 4, 5, 6) by using the corresponding anatomical structures previously segmented. From numerical and visual results, we can notice that even if minor corresponding segmented elements are missing in volume pairs, our method is able to reduce the initial registration errors. However, in the case of volumes acquired after removal, resection cavities may be segmented by our method due to their intensity similar to the sulci. Consequently, the mTRE in Table 5 is reduced less with respect to Table 4, since these structures have no or few corresponding elements in volumes acquired in previous steps. This is a limiting factor of our registration method, which is completely based on the masks generated by our trained model. In future work, we could try to segment such structures and exclude them during the registration. Only another work [25] focused on the registration of US volumes acquired before and after resection of RESECT dataset (Table 5). The mTRE obtained by the aforementioned approach is better than our method, which, however, is the first one to provide results for the volumes obtained before and during resection of the RESECT dataset. In this set of volumes, our registration performs quite well, reducing the initial mTRE to 1.36 mm.

Regarding the BITE dataset, our algorithm improves the initial registration (see Table 6), proving not to be over-tuned on RESECT dataset. Note that in contrast to our approach, all other methods compared in Table 6 have only been tested on the BITE dataset. Thus, the results may be over-tuned on this limited set of volumes and the approaches could lack generalization. On the contrary, our solution is the second one after [25] to propose a more generalized method, which has been tested on registering the volumes of both RESECT and BITE datasets. Therefore, our method is validated on a larger number of US acquisitions, providing a more generalized solution. Nevertheless, there might be some reasons why a few other approaches have smaller average mTREs for the BITE dataset (last section of Table 6). First of all, a numerical impacting factor for our results comes from case 12, where the TRE increases from 10.54 up to 11.08 mm, affecting the overall result. The capture range of our method is too low to register this volumes pair, which has a very large initial misalignment. In future work, we could improve the results by performing an initial registration which could increase the capture range of our method. Moreover, the limited improvement obtained by our method might be due to the lower quality of the BITE dataset with respect to the RESECT volumes, which is used for training the segmentation approach. Since our registration method is based on the generated masks, it is almost impossible for the registration method to converge to the right solution if the segmented masks are not accurate enough.

The total time required by each task of our method is visible in Table 3: The segmentation step requires 1.28 s and 28.55 s (before/during) and 29.40 s (during/after) that are needed to register the generated 3D masks. In addition to this, we should also take into account the time to reconstruct a 3D US volumes from 2D images, which is of a few seconds [14]. Considering the increase in the brain shift over the time and the average duration of a neurosurgical procedure [34], our algorithm is fast enough to register US volumes and therefore provides a meaningful solution for brain shift. Nevertheless, in future work we could optimize our algorithm in order to speed up the registration step.

## Conclusion

To the best of our knowledge, our solution is the first one to propose a segmentation-based registration method which registers US volumes acquired at different surgical stages. Our approach provides some important contributions. Regarding the segmentation step, a model based on a 3D U-Net has been trained on a large number of anatomical structures, whose manual annotations have been validated by an experienced neurosurgeon. Even if the training is performed on a sparse set of annotations, the proposed solution is able to automatically segment hyperechogenic elements in US volumes. Moreover, the segmented anatomical structures prove to be meaningful elements which can guide the registration of US volumes acquired in the neurosurgical context. In fact, for two different datasets of US volumes acquired at different surgical stages, the initial mTREs are correctly reduced, demonstrating that our solution is not over-tuned for a specific dataset. Moreover, our work is the first one to be applied also on the US volumes of RESECT dataset acquired during resection, for which no previous work has been published.

## Footnotes

1 Surgicel (Ethicon, Somerville, NJ).

2 https://www.mevislab.de/mevislab/.
